# Gynæcology and Obstetrics

**Published:** 1906-01-06

**Authors:** 


### GYNÆCOLOGY AND OBSTETRICS.

**Ectopic Pregnancy.**—The chief clinical features of this condition have been recently set forth in a lucid lecture by Dr. Lewers.[1] Since Lawson Tait performed his first laparotomy for ruptured tubal pregnancy in 1883, it has become clear that ectopic pregnancy is, in reality, comparatively common. In former years such cases were classed as *pelvic hæmatocele*, but it is now known, as the result of Tait's work, that the effusion of blood is caused either by a tubal abortion or by the rupture of a gravid tube. Failure to find a fœtus or even an amniotic cavity in such a mass is not conclusive evidence against pregnancy. Mciroscopic sections must be made to demonstrate the presence of chorionic villi, which are readily recognised. Ectopic pregnancy—and Lewers prefers this name to the term extrauterine, as it includes all cases in which the ovum does not develop in the normal cavity of the uterus—is extremely rare in the second half of gestation. The ovum is practically always situated in the Fallopian tube. A primary ovarian pregnancy has been observed, but is so rare as to be a pathological curiosity. A primary abdominal pregnancy, although a theoretical possibility, has not yet been actually demonstrated. Occasionally pregnancy occurs in the rudimentary horn of a bicornuate uterus. In the tube it is most commonly situated in the outer portion, less often in the isthmus, and still less frequently in the interstitial portion which passes through the uterine wall. In all cases in which a fœtus is developing outside the normal uterine cavity the uterus hypertrophies, and the nearer the ovum develops to the uterus the more marked is the enlargement. The fertilised ovum fixes itself in the tube by a method similar to that which it is now known to adopt in the uterus—that is to say, the peripheral cells of the ovum, the trophoblast, burrow into the mucous membrane and erode it, thus getting beneath it, and so coming to be outside the lumen of the tube. This active erosion of the tubal wall by the cells of the trophoblast opens up large vessels and leads to hæmorrhages which increase the bulk of the ovum, and so assist in expediting the termination of the pregnancy either by tubal abortion or rupture. Tubal abortion—that is, expulsion of the ovum into and out of the dilated and contracting tube, occurs in three-fourths of the cases of tubal pregnancy, and is a relatively favourable termination. If the abortion is complete, and the whole of the ovum is extruded into the peritoneal cavity, it dies, and hæmorrhage may not be severe. Usually, however, it is only partially extruded, and bleeding continues until the ovum is either wholly expelled or removed. In the still more serious event of tubal rupture, which occurs in about one-fourth of the cases, the tube ruptures and the ovum is expelled through the rent. If the rupture occurs in a part of the tube covered by peritoneum the fœtus lies free in the peritoneal cavity, but the placenta is generally partially or wholly retained in the tube. Free bleeding takes place into the peritoneum from the vessels torn across by the rupture, and also from those opened up by the erosion of the trophoblast, and the mother may die from internal hæmorrhage. The fœtus usually dies, but if the mother survives, and the placenta is not much interfered with, it may go on developing. Very rarely rupture may take place, not through the peritoneum, but into the space between the layers of the broad ligament, the fœtus dies, and a hæmatoma of the broad ligament is formed, which, however, cannot often be recognised as such clinically. If the placenta is not much interfered with the fœtus may continue to grow, and by its growth gradually strip up the peritoneum. Rupture into the broad ligament tissues is very favourable for the mother, as but little bleeding can take place into the confined space. As a good working rule, one may consider that tubal pregnancy will terminate by a more or less grave crisis within about 12 weeks of conception. As regards etiology and symptoms a preceding interval of actual or relative sterility is common; either the patient has been married some years but has not been pregnant, or she has not had a child recently. In a typical case the patient, having previously menstruated regularly, misses a period, and, after an interval of five or six weeks, gets pains in one or other iliac fossa, accompanied by slight but persistent hæmorrhage, which frequently takes the form of a brownish-red discharge. Then suddenly one day she is seized with severe pain in the lower abdomen, and becomes acutely ill with faintness, collapse, and marked pallor, signs of hæmorrhage which must be internal since there is no sufficient external cause. Such a picture is very characteristic, and justifies immediate operation, although in one such case observed by Lewers the condition proved to be not tubal pregnancy, but pyosalpinx. Operation is usually simple, and consists in opening the abdomen, drawing up the tube, transfixing it and usually removing the tube and ovary. Where collapse and blanching are severe a preliminary saline infusion is desirable. In the much less grave cases in which a pelvic hæmatocele forms, due generally to tubal abortion, it may be permissible to defer operation and await natural absorption if the patient can be kept under conditions suitable for immediate operation shoul dfresh hæmorrhage occur. It is not possible to foretell in which cases hæmorrhage will recur or in which absorption will take place. Tubal pregnancy may be followed by normal pregnancy; in fact, the normal condition appears to be the rule in those cases in which another pregnancy succeeds; hence Mr. Alban Doran [2] concludes that it is not justifiable to amputate both tubes for the first occurrence, although repeated tubal pregnancy does occur. J. C. H. Leicester [3] has reported a case of tubal pregnancy which apparently ruptured twice; but possibly the severe pain first noted might be explained by the erosion of the trophoblast leading to hæmorrhage, as described by Lewers.

[1] Clin. Jour., Oct. 4. [2] Lancet, June 17, p. 1648. [3] Ibid., Oct. 14, p. 1110.

